# Effectiveness of Ginsenoside Rh2 Treatment in Mice Experimentally Infected With *Cryptosporidium parvum*

**DOI:** 10.1002/vms3.71133

**Published:** 2026-08-09

**Authors:** Esengul Yildirim, Engin Balikci

**Affiliations:** ^1^ Department of Veterinary Internal Medicine Faculty of Veterinary Medicine Fırat University Elazığ Turkey

**Keywords:** *Cryptosporidium parvum*, ginsenoside, halofuginone, murine, oocyst

## Abstract

**Objective:**

To evaluate the therapeutic potential of Ginsenoside Rh2 in mice experimentally infected with *Cryptosporidium parvum* and to compare the efficacy of two Ginsenoside Rh2 dosing regimens.

**Animals:**

Fifty male BALB/c mice, aged four weeks, were used.

**Methods:**

Mice were immunosuppressed with dexamethasone and orally infected with 3 × 10^5^ *C. parvum* oocysts. Animals were randomly assigned to five groups (*n* = 10): healthy control (HC), infected control (IC), halofuginone‐treated (HAL), low‐dose Ginsenoside Rh2 (GIN50) and high‐dose Ginsenoside Rh2 (GIN100). Mice were treated for 5 days, and body weights and oocyst counts were recorded at several time points. On Day 18 (T18), mice were euthanized, and intestinal samples were collected for histopathology.

**Results:**

The HAL, GIN50 and GIN100 groups showed significant body‐weight gains, with the GIN100 group exhibiting the greatest increase from T0 to T18 (16.70 ± 0.14 g to 24.00 ± 0.24 g, *p* < 0.001). Oocyst counts decreased significantly in all treated groups over time. In the GIN100 group, oocyst counts decreased from 3.69 ± 0.01 at T0 to 0.99 ± 0.03 at T5 (*p* < 0.001), and no oocysts were detected at T18. Group‐level histopathological assessment showed the least lesion severity in the HAL group, whereas GIN50 and GIN100 showed comparable mild lesion scores.

**Conclusion:**

Ginsenoside Rh2, particularly at 100 mg/kg, reduced *C. parvum* oocyst shedding and improved body‐weight recovery in experimentally infected mice. However, its comparative efficacy, safety profile and mechanism of action require further investigation before it can be considered an established alternative treatment.

## Graphical abstract

• This study evaluated the therapeutic effects of Ginsenoside Rh2 in mice experimentally infected with *Cryptosporidium parvum* and compared two dosing regimens.

• Ginsenoside Rh2 reduced faecal oocyst shedding and improved body‐weight recovery in *C. parvum*‐infected mice.

• The high‐dose Rh2 regimen (100 mg/kg) showed the most pronounced parasitological and body‐weight response among the Rh2‐treated groups.

• Histopathological improvement was observed in treated groups, although halofuginone showed the most pronounced mucosal recovery.

• Ginsenoside Rh2 may be considered a candidate anti‐cryptosporidial compound requiring further investigation of its efficacy, safety, optimal dose and mechanism of action.

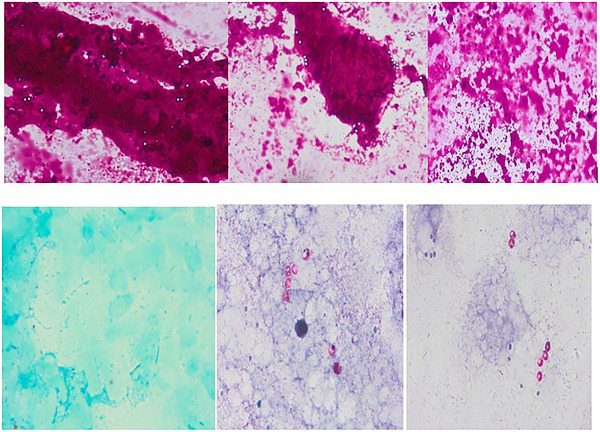

## 1 Introduction

*Cryptosporidium parvum* is an obligate intracellular protozoan parasite belonging to the phylum Apicomplexa. It is an important cause of enteric disease and waterborne diarrheal outbreaks in humans and animals worldwide (Bouzid et al. 2013). Transmission occurs primarily through the faecal–oral route via ingestion of oocysts that are highly resistant to environmental stressors and common water treatment methods such as chlorination (Efstratiou et al. 2017). Once ingested, the oocysts release sporozoites that invade the epithelial cells of the small intestine, leading to disruption of the intestinal barrier, malabsorption and secretory diarrhoea (Laurent et al. 1999). Nitazoxanide is the only drug approved by the US Food and Drug Administration for the treatment of cryptosporidiosis in humans; however, its efficacy is limited in immunocompromised patients, and therapeutic options remain limited in veterinary and immunosuppressed settings (Ali et al. 2024). In recent years, natural compounds have gained attention as potential antiparasitic candidates because of their broad biological activities; however, their efficacy and safety require compound‐specific experimental validation (Ali and Mishra 2024).

Ginsenoside Rh2, a secondary metabolite derived from Panax ginseng, has been investigated for its anticancer, anti‐inflammatory and immunomodulatory properties (He et al. 2022; Li et al. 2020). Its biological effects have been associated with modulation of innate immune responses, including TLR4/NF‐κB‐related signalling pathways (Pan et al. 2024). In addition, an in vitro study reported that Ginsenoside Rh2 inhibited the intracellular development of *C. parvum* in a dose‐dependent manner (Shahiduzzaman et al. 2020). However, evidence regarding the anti‐*Cryptosporidium* activity of Ginsenoside Rh2 remains limited to in vitro observations, and its potential therapeutic effect has not been evaluated in an experimental in vivo infection model.

A comprehensive literature search conducted in May 2025 using Web of Science, PubMed, Scopus and Google Scholar revealed no in vivo studies evaluating the effects of Ginsenoside Rh2 on *C. parvum* infection. Therefore, the primary aim of this study was to evaluate the therapeutic potential of Ginsenoside Rh2 in mice experimentally infected with *C. parvum*. A secondary objective was to compare two dosing regimens of Ginsenoside Rh2, administered at 50 and 100 mg/kg/day. We hypothesized that Ginsenoside Rh2 would reduce oocyst shedding and improve infection‐associated body‐weight and histopathological outcomes in this experimental model.

## 2 Materials and Methods

The present study was conducted at the Animal Experiment Unit of the Erzurum Veterinary Control Institution, Erzurum, Turkey. All procedures were performed in accordance with the guidelines outlined in the National Institutes of Health Guide for the Care and Use of Laboratory Animals. The experimental protocol was approved by the Local Ethics Committee for Animal Experiments of the Erzurum Veterinary Control Institution (Approval No: 74/2022). Prior to infection with *C. parvum*, the mice were allowed to acclimate to the experimental conditions for 10 days. The mice were housed in groups of 10 in well‐ventilated plastic cages with clean wood‐chip bedding. All mice were maintained at a controlled temperature (24  ± 1°C) under a 12/12 h light/dark cycle and provided ad libitum access to a standard diet containing 24% protein, 4% fat, approximately 4%–5% fibre and water.

### 2.1 Collection and Preparation of *C. parvum* Oocysts

*C. parvum* oocysts were naturally collected from the faeces of infected calves, as described previously (Aboelsoued et al. 2019). Faecal samples from diarrheic calves were initially tested for *C. parvum* and other pathogens using a rapid test kit (Bionote, Hwaseong‐si, Republic of Korea). After confirming the absence of *Eimeria spp*. and other parasitic agents through microscopic examination, the faeces were diluted with distilled water, filtered through 40 µm mesh filters and mixed with a saturated NaCl solution to concentrate the oocysts. The mixture was centrifuged at 1000 × *g* for 10 min at 4°C. The supernatant was discarded, and the sediment was resuspended in distilled water (1:3 dilution). After centrifugation at 4000 × *g* for 10 min, the sediment was stored in 2.5% potassium dichromate solution at 4°C until inoculation. Prior to infection, potassium dichromate was removed by centrifugation, and oocyst concentration was adjusted. Oocyst concentrations were determined by counting the average number of oocysts per 1 mL of sample (Madbouly Taha et al. 2017), and slides were prepared using modified Ziehl–Neelsen staining.

### 2.2 Inoculation of *C. parvum* Oocysts

Mice were immunosuppressed with dexamethasone (0.25 µg/g/day for 7 days) in drinking water, as previously described (Ren et al. 2012). They were then infected orally with approximately 3 × 10^5^ *C. parvum* oocysts via gastric gavage (Madbouly Taha et al. 2017). Parasitic examination commenced 18 h post‐inoculation, and faecal samples were collected every other day for 7 days for microscopic examination. Infection was confirmed when oocyst concentrations in faeces ranged from 2–5 × 10^4^ oocysts per sample, identified through carbolfuchsin staining.

### 2.3 Groups

A total of 50 four‐week‐old male BALB/c mice (approximately 20 ± 0.5 g) were randomly assigned to five groups (*n* = 10 per group): healthy control (HC; uninfected and untreated), infected control (IC; infected but untreated), halofuginone‐treated group (HAL; infected and treated with halofuginone lactate at 100 µg/kg/day; Halocur, Intervet, France), low‐dose Ginsenoside Rh2 group (GIN50; infected and treated with Ginsenoside Rh2 at 50 mg/kg/day for five consecutive days; Bostonchem, Boston, USA) and high‐dose Ginsenoside Rh2 group (GIN100; infected and treated with Ginsenoside Rh2 at 100 mg/kg/day for five consecutive days). The doses of halofuginone lactate and Ginsenoside Rh2 were selected based on previous studies assessing their effectiveness in parasitic infections (Xu et al. 2020; Valigurová et al. 2018). Mice's body weights were recorded prior to each treatment, on the final day of immunosuppressive agent administration (T0), and on Days 1 (T1), 2 (T2), 3 (T3), 4 (T4), 5 (T5) and 18 (T18) following the initiation of treatment.

### 2.4 Parasitological Evaluation

Stool samples were preserved in 2.5% potassium dichromate and processed as previously described (Nalbantoğlu 2007). Oocysts were identified using carbolfuchsin staining and counted using the modified Ziehl–Neelsen technique, with oocysts enumerated in 10 microscopic fields at 100× magnification (Villacorta et al. 1991). Oocyst counts were performed on T0, T1, T2, T3, T4, T5 and T18. Oocyst counts were expressed as the number of oocysts per 50 µL of faecal suspension before logarithmic transformation for statistical analysis.

### 2.5 Histopathological Examination

On T18, animals were euthanized, necropsied and intestinal samples were collected from the duodenum, jejunum and ileum for histopathological examination. Tissues were fixed in 10% neutral‐buffered formalin, processed for histology and stained with haematoxylin‐eosin. The severity of *C. parvum*‐associated intestinal lesions was evaluated semi‐quantitatively using a 0–3 grading system. Grade 0 indicated no detectable cryptosporidia on the mucosal surface and no apparent pathological change; Grade 1 indicated mild infection characterized by isolated or sporadic cryptosporidia with minimal epithelial alteration; Grade 2 indicated moderate infection with more widely distributed cryptosporidia, villous degeneration/blunting and inflammatory cell infiltration; and Grade 3 indicated severe infection characterized by extensive epithelial surface involvement, marked villous shortening/blunting, epithelial disruption and prominent inflammatory cell infiltration (Koudela and Jiří 1997).

### 2.6 Statistical Analysis

Statistical analyses were performed using IBM SPSS Statistics for Windows, version 25.0 (IBM Corp., Armonk, NY, USA). Data are presented as mean ± standard error. Oocyst count data were logarithmically transformed before analysis to reduce skewness and improve distributional assumptions. Repeated‐measures ANOVA was used to evaluate within‐group changes over time for body weight and log‐transformed oocyst counts. Mauchly's test was used to assess the sphericity assumption. When the sphericity assumption was violated, Greenhouse–Geisser or Huynh–Feldt corrections were applied, as appropriate. Bonferroni adjustment was used for pairwise comparisons over time. Between‐group comparisons at each time point were performed using one‐way ANOVA followed by Tukey's honestly significant difference post hoc test. Semi‐quantitative histopathological lesion scores were summarized descriptively as group‐level scores. Therefore, no separate inferential statistical analysis was performed for histopathological lesion scores. A value of *p* < 0.05 was considered statistically significant.

## 3 Results

From T0 to T18, the HC group exhibited a significant increase in body weight, rising from 22.20 ± 0.09 g to 23.00 ± 0.09 g (*p* < 0.001). In contrast, the IC group showed a significant decrease from 15.30 ± 0.19 g to 15.00 ± 0.09 g (*p* < 0.001). The HAL, GIN50 and GIN100 groups all demonstrated significant weight gains over the same period, with the GIN100 group showing the most pronounced increase, from 16.70 ± 0.14 g to 24.00 ± 0.24 g (*p* < 0.001) (Table 1).

**TABLE 1:** Body weight changes of BALB/c mice during the treatment period and at T18. A total of 50 male BALB/c mice, aged 4 weeks, were randomly assigned to five groups (*n* = 10 each): Healthy control (HC; uninfected and untreated), infected control (IC; infected and untreated), halofuginone‐treated group (HAL; infected and treated with halofuginone lactate at 100 µg/kg/day), low‐dose Ginsenoside Rh2 group (GIN50; infected and treated with Ginsenoside Rh2 at 50 mg/kg/day) and high‐dose Ginsenoside Rh2 group (GIN100; infected and treated with Ginsenoside Rh2 at 100 mg/kg/day). Body weights were recorded at treatment initiation (T0), during treatment (T1–T5), and at the final evaluation point (T18). Data are presented as mean ± standard error.

Groups	Days							*p*
	T0	T1	T2	T3	T4	T5	T18	
HC	22.20 ± 0.09^D, a^	22.30 ± 0.08^C, a^	22.30 ± 0.09^CD, a^	22.45 ± 0.07^CD, a^	22.56 ± 0.07^B, a^	22.70 ± 0.08^AB, a^	23.00 ± 0.09^A, b^	< 0.001
IC	15.30 ± 0.19^A, c^	14.90 ± 017^AB, d^	14.70 ± 016^B, d^	14.50 ± 0.15^BC, d^	14.50 ± 0.15^BC, d^	14.50 ± 0.15^BC, c^	15.00 ± 0.09^AB, e^	< 0.001
HAL	16.70 ± 0.07^F, b^	17.00 ± 0.07 ^E, c^	17.80 ± 0.09^D, c^	18.00 ± 0.08^D,c^	18.90 ± 0.09^C, c^	19.80 ± 0.07^B, b^	21.00 ± 0.20^A, d^	< 0.001
GIN50	16.70 ± 0.07^F, b^	17.00 ± 0.07^E, c^	17.80 ± 0.12^D, c^	18.00 ± 0.11^D, c^	18.90 ± 0.08^C, c^	20.00 ± 0.16^B, b^	22.00 ± 0.36^A, c^	< 0.001
GIN100	16.70 ±0.14^F, b^	18.60 ± 0.07^E, b^	19.00 ± 0.10^D, b^	19.30 ± 0.05^CD, b^	19.50 ± 0.06^C, b^	19.90 ± 0.08^B, b^	24.00 ± 0.24^A, a^	< 0.001

*Note*: Different uppercase superscript letters within the same row indicate significant differences among time points within the same group. Different lowercase superscript letters within the same column indicate significant differences among groups at the same time point. Values sharing the same superscript letter are not significantly different. Statistical significance was accepted at *p* < 0.05.

Oocyst counts in the IC group increased significantly from 3.70 ± 0.01 at T0 to 5.63 ± 0.09 at T18 (*p* < 0.001), indicating progressive infection. In contrast, the HAL, GIN50 and GIN100 groups showed significant reductions in oocyst counts over time. In the HAL group, oocyst counts declined from 3.67 ± 0.02 at T0 to 1.04 ± 0.04 at T5 (*p* < 0.001) and no oocysts were detected at T18. Similarly, the GIN50 group showed a significant decrease from 3.67 ± 0.01 at T0 to 1.09 ± 0.03 at T5 (*p* < 0.001), with no detectable oocysts at T18. The most pronounced numerical reduction at T5 was observed in the GIN100 group, where oocyst counts decreased from 3.69 ± 0.01 at T0 to 0.99 ± 0.03 at T5 (*p* < 0.001) and no oocysts were detected at T18 (Table 2). Based on the log‐transformed mean values presented in Table 2, oocyst counts at T5 were reduced by 80.2% in HAL, 79.2% in GIN50 and 81.1% in GIN100 relative to the IC group. At T18, no oocysts were detected in any of the treated groups.

**TABLE 2:** Oocyst counts in faecal samples of infected BALB/c mice during the treatment period and at T18. Oocyst counts were determined per 50 µL of faecal suspension and log‐transformed before statistical analysis. Experimental groups included infected control (IC; infected and untreated), halofuginone‐treated group (HAL; infected and treated with halofuginone lactate at 100 µg/kg/day), low‐dose Ginsenoside Rh2 group (GIN50; infected and treated with Ginsenoside Rh2 at 50 mg/kg/day) and high‐dose Ginsenoside Rh2 group (GIN100; infected and treated with Ginsenoside Rh2 at 100 mg/kg/day). Oocyst counts were recorded at treatment initiation (T0), during treatment (T1–T5), and at the final evaluation point (T18). Data are presented as mean ± standard error.

Groups	Days							*p*
	T0	T1	T2	T3	T4	T5	T18	
IC	3.70 ± 0.01^G, a^	3.77 ± 0.01^F, ab^	3.98 ± 0.01^E, a^	4.27 ± 0.001^D, a^	4.51 ± 0.01^C, a^	5.24 ± 0.01^B, a^	5.63 ± 0.01^A^	< 0.001
HAL	3.67 ± 0.02^B, a^	3.75 ± 0.01^A, bc^	3.47 ± 0.02^C, b^	2.65 ± 0.02^D, b^	2.14 ± 0.03^E, b^	1.04 ± 0.04^F, b^	ND	< 0.001
GIN50	3.67 ± 0.01^B, a^	3.75 ± 0.01^A, c^	3.49 ± 0.01^C, b^	2.69 ± 0.02^D, b^	2.16 ± 0.03^E, b^	1.09 ± 0.03^F, b^	ND	< 0.001
GIN100	3.69 ± 0.01^B, a^	3.78 ± 0.01^A, a^	3.44 ± 0.02^C, b^	2.67 ± 0.02^D, b^	2.08 ± 0.04^E, b^	0.99 ± 0.03^F, b^	ND	< 0.001

*Note*: Different uppercase superscript letters within the same row indicate significant differences among time points within the same group. Different lowercase superscript letters within the same column indicate significant differences among groups at the same time point. Values sharing the same superscript letter are not significantly different. Statistical significance was accepted at *p* < 0.05. ND indicates that no oocysts were detected in the examined faecal samples at T18.

Semi‐quantitative group‐level histopathological lesion scores are presented in Table 3. The HC group showed no detectable cryptosporidia on the mucosal surface and no pathological changes, corresponding to a lesion score of 0. The IC group showed the most severe intestinal lesions, including extensive *C. parvum* colonization, villous shortening/blunting, epithelial disruption and inflammatory cell infiltration and was assigned a lesion score of 3. Compared with the IC group, the HAL, GIN50 and GIN100 groups showed lower lesion severity, with fewer residual *Cryptosporidium* forms, reduced mucosal damage and evidence of epithelial regeneration. These treated groups were assigned a group‐level lesion score of 1. The histopathological findings were broadly comparable between GIN50 and GIN100 at the group‐score level (Figure 1).

**TABLE 3:** Semi‐quantitative group‐level histopathological lesion scores of the small intestine at T18. Experimental groups included: Infected control (IC; infected, untreated), halofuginone‐treated (HAL; infected, treated with halofuginone lactate at 100 µg/kg/day), low‐dose Ginsenoside Rh2 (GIN50; infected, treated with 50 mg/kg/day) and high‐dose Ginsenoside Rh2 (GIN100; infected, treated with 100 mg/kg/day).

Group	Histopathological finding	Lesion severity	Group‐level lesion score
HC	No cryptosporidia on the mucosal surface; no pathological change	None	0
IC	Extensive *Cryptosporidium* colonization with severe villous shortening/blunting and mononuclear inflammatory cell infiltration	Severe	3
HAL	Few residual *Cryptosporidium* forms with reduced mucosal damage and epithelial regeneration	Mild to moderate	1
GIN50	Sporadic *Cryptosporidium* forms with partial villous degeneration/blunting and epithelial regeneration	Mild	1
GIN100	Sporadic developmental forms of *Cryptosporidium* with partial villous degeneration/blunting and epithelial regeneration	Mild	1

*Note*: Scores were based on a semi‐quantitative 0–3 grading system: 0 = no pathological change/no detectable cryptosporidia on the mucosal surface; 1 = mild lesion; 2 = moderate lesion; 3 = severe lesion. Histopathological lesion scores were summarized descriptively at the group level; therefore, no separate inferential statistical comparison was performed for this outcome.

**FIGURE 1:**
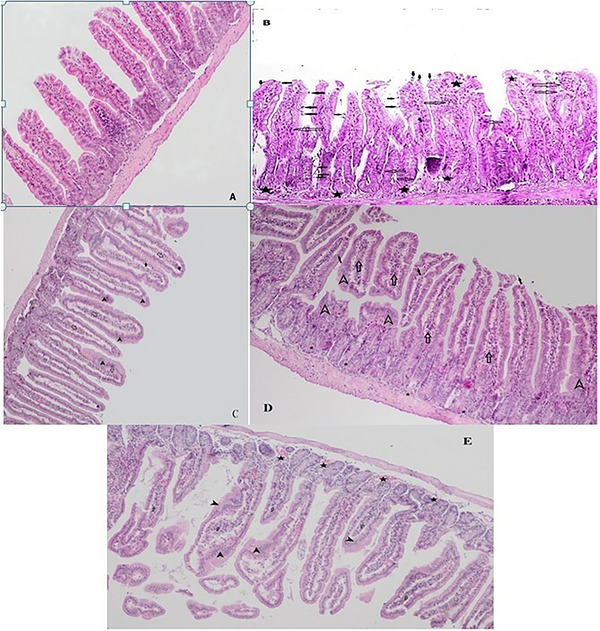
Representative histopathological findings of the small intestine at T18. (A) Healthy control group: normal intestinal mucosal architecture without detectable *Cryptosporidium* forms or pathological lesions. (B) Infected control group: severe intestinal lesions characterized by mature *Cryptosporidium* forms on the villous surface, developmental stages of *Cryptosporidium*, villous degeneration/blunting, inflammatory cell infiltration in the lamina propria, intraepithelial lymphocytes, crypt epithelial degeneration/atrophy and epithelial disruption associated with parasite attachment. (C) Halofuginone‐treated group: few residual mature *Cryptosporidium* forms, inflammatory cell infiltration in the lamina propria, intraepithelial lymphocytes, crypt epithelial regeneration and increased columnar epithelial cells. (D) Ginsenoside Rh2 50 mg/kg group: residual mature *Cryptosporidium* forms and developmental stages of *Cryptosporidium* with partial villous degeneration/blunting, inflammatory cell infiltration, intraepithelial lymphocytes, crypt epithelial regeneration and increased columnar epithelial cells. (E) Ginsenoside Rh2 100 mg/kg group: residual developmental stages of *Cryptosporidium* with partial villous degeneration/blunting, inflammatory cell infiltration, intraepithelial lymphocytes, crypt epithelial regeneration and increased columnar epithelial cells. Black arrows indicate mature *Cryptosporidium* forms; black stars indicate developmental stages of *Cryptosporidium*; white arrows indicate intraepithelial lymphocytes; and arrowheads indicate increased columnar epithelial cells/epithelial regeneration. Haematoxylin and eosin staining. Scale bars: A = 20 µm, B = 20 µm, C = 10 µm, D = 20 µm and E = 10 µm.

## 4 Discussion

This study indicates that Ginsenoside Rh2 may have therapeutic potential in mice experimentally infected with *C. parvum*, as reflected by reduced oocyst shedding, improved body‐weight recovery and partial histological improvement. However, the comparative efficacy of Rh2 should be interpreted cautiously and according to each outcome. GIN100 produced the most pronounced reduction in oocyst shedding and the greatest body‐weight recovery among the Rh2‐treated groups, whereas GIN50 and GIN100 showed broadly comparable group‐level histopathological lesion scores. In contrast, HAL appeared to provide the most pronounced histological recovery. Therefore, GIN100 should not be interpreted as globally superior to GIN50 or HAL; rather, its advantage was mainly observed in parasitological and body‐weight outcomes. These findings are consistent with previous reports describing the antiparasitic potential of ginsenosides and other natural or semi‐synthetic compounds (Shahiduzzaman et al. 2020; Guo et al. 2018; Aydogdu et al. 2018). Nevertheless, Ginsenoside Rh2 should be regarded as a candidate anti‐cryptosporidial compound requiring further investigation, rather than as an established alternative treatment.

Mice in the IC group showed progressive weight loss and increasing oocyst counts, reflecting the detrimental effects of *C. parvum* infection on intestinal absorption and host condition. In contrast, all treated groups demonstrated improved body‐weight recovery and marked reductions in oocyst shedding. No oocysts were detected at T18 in any of the treated groups, indicating a marked reduction in faecal oocyst shedding after treatment. However, because HAL, GIN50 and GIN100 all reached non‐detectable oocyst levels at T18, differences among treated groups should be interpreted primarily using earlier treatment time points, particularly T5, together with body‐weight recovery and histopathological findings. Histopathological findings in the IC group were consistent with typical cryptosporidial intestinal lesions, including villous atrophy, epithelial damage and inflammation (Hassan et al. 2021; El‐Wakil et al. 2021). Based on the group‐level histopathological lesion scores, the treated groups showed lower lesion severity than the IC group. Among the treated groups, HAL appeared to provide the most pronounced mucosal recovery, whereas GIN50 and GIN100 showed comparable group‐level histopathological scores. Possible spontaneous recovery by T18 should also be considered when interpreting the final oocyst findings. However, oocyst counts continued to increase in the untreated IC group up to T18, suggesting persistent infection under the present immunosuppressed experimental conditions. Therefore, the absence of detectable oocysts in the treated groups at T18 is unlikely to be explained solely by spontaneous recovery, although this possibility cannot be completely excluded without additional untreated recovery or longer follow‐up groups.

The mechanism underlying the observed effects of Ginsenoside Rh2 could not be determined in the present study because inflammatory, oxidative stress, immunological and molecular biomarkers were not evaluated. Previous in vitro evidence has shown that Ginsenoside Rh2 can inhibit the intracellular development of *C. parvum*, suggesting a possible direct anti‐cryptosporidial effect (Shahiduzzaman et al. 2020). In addition, Rh2 has been associated with anti‐inflammatory and immunomodulatory activity, including modulation of TLR4/NF‐κB‐related signalling pathways (Pan et al. 2024). Other studies have reported that ginsenosides may affect pathogen–host cell interactions, including mechanisms related to cell‐surface receptors (Dong et al. 2017), and that Rh2 can modulate intracellular signalling pathways involved in cell survival, apoptosis and autophagy (Li et al. 2019). Host‐cell signalling mechanisms, including PI3K‐related pathways, have also been implicated in *C. parvum* invasion and intracellular development (Chen et al. 2004). Moreover, immune mediators such as TNF‐α and IFN‐γ are involved in host responses to *C. parvum* infection (McDonald et al. 2013), and Rh2 has been reported to modulate immune responses in experimental models (Wang et al. 2017). Therefore, the beneficial effects observed in the present study may reflect direct effects on parasite development, modulation of host inflammatory or immune responses, or a combination of both. However, because these pathways were not directly measured, this interpretation remains speculative.

Safety should also be interpreted cautiously in the present study because no dedicated toxicological evaluation was performed. The study did not include haematological or serum biochemical analyses, liver and kidney histopathology or long‐term follow‐up. Therefore, the present findings cannot establish the safety profile of Ginsenoside Rh2. Future studies should investigate the pharmacodynamics, optimal dosing, dose‐dependent safety and biomarker‐based mechanisms of Rh2 in immunocompromised models, as *Cryptosporidium* infection is particularly severe in individuals with weakened immune systems (Madbouly Taha et al. 2017). The use of dexamethasone‐induced immunosuppression should also be considered when interpreting the present findings. Dexamethasone was used to facilitate the establishment and persistence of *C. parvum* infection, as described in previous experimental models (Madbouly Taha et al. 2017; Ren et al. 2012). However, immunosuppression may alter host immune responses, parasite replication dynamics, faecal oocyst shedding, intestinal lesion development and treatment response. In the present study, immunosuppression was induced according to a previously described protocol, but it was not independently confirmed using leucocyte counts, lymphocyte profiling, cytokine measurements or other immune markers. Therefore, the results should be interpreted within the context of an experimentally immunosuppressed murine model and should not be directly extrapolated to immunocompetent animals, other host species, or natural infection conditions without further validation.

This study has several limitations. First, the findings were obtained from an experimentally immunosuppressed murine model; therefore, they should not be directly extrapolated to immunocompetent animals, other host species or natural infection conditions without further validation. Second, although halofuginone lactate was included as a positive comparator because of its use in veterinary and experimental anti‐cryptosporidial settings, a nitazoxanide‐treated group was not included. Therefore, the efficacy of Ginsenoside Rh2 relative to nitazoxanide remains to be evaluated. Third, systematic toxicological assessments, including haematological and serum biochemical analyses, liver and kidney histopathology and long‐term follow‐up, were not performed; therefore, the safety and long‐term tolerability of Rh2 remain uncertain. Fourth, the study did not include individual animal‐level histopathological scoring, inflammatory or oxidative stress biomarkers or molecular analyses; therefore, the mechanisms underlying the observed effects could not be determined. Finally, although two Rh2 doses were evaluated, the optimal dosing regimen remains to be established.

In conclusion, this study indicates that Ginsenoside Rh2, particularly at 100 mg/kg, reduced *C. parvum* oocyst shedding and improved body‐weight recovery in experimentally infected mice. HAL appeared to provide the most pronounced histological recovery, whereas GIN100 showed the most pronounced parasitological and body‐weight response among the Rh2‐treated groups. Because dedicated toxicological and mechanistic assessments were not performed, Ginsenoside Rh2 should be considered a candidate anti‐cryptosporidial compound requiring further investigation rather than an established alternative treatment. Future studies should confirm its comparative efficacy, safety profile and mechanism of action using individual‐level histopathological scoring, biomarker assessment and dose‐dependent toxicological evaluation.

## Author Contributions

**Esengül Yildirim**: conceptualization, methodology, data curation, formal analysis, writing – original draft. **Engin Balikci**: conceptualization, writing – review and editing, supervision.

## Funding

The study was financially supported by FUBAP (project number VF.21.22).

## Ethics Statement

The study protocol was carried out after approved by the Local Ethics Committee for Animal Experiments of the Erzurum Veterinary Control Institution (Decision No: 74/2022).

## Conflicts of Interest

The authors declare no conflicts of interest.

## Data Availability

The data that support the findings of this study are available on request from the authors.
